# Factors Influencing Evidence-Based Practice Engagement Among Clinical Dietitians: A Mixed-Methods Study Using the COM-B Model

**DOI:** 10.3390/healthcare14070893

**Published:** 2026-03-31

**Authors:** Abeer Almudaihim

**Affiliations:** 1 Department of Clinical Nutrition, College of Applied Medical Sciences, King Saud bin Abdulaziz University for Health Sciences, Riyadh 11481, Saudi Arabia; mudaiheema@ksau-hs.edu.sa; 2 King Abdullah International Medical Research Center, Riyadh 11481, Saudi Arabia; 3 Department of Clinical Nutrition, Ministry of National Guard Health Affairs, Riyadh 11426, Saudi Arabia

**Keywords:** evidence-based practice, clinical dietitians, COM-B model, behavioral determinants, Saudi Arabia, mixed methods, capability, motivation, opportunity

## Abstract

**Highlights:**

**What are the main findings?**

- Reflective motivation and physical and social opportunity were the strongest determinants of evidence-based practice (EBP) engagement among clinical dietitians.
- Psychological capability was high but did not independently predict routine EBP use.

**What are the implications of the main findings?**

- Interventions to improve EBP should extend beyond knowledge and address motivational and organizational factors.
- Healthcare organizations play a key role in enabling EBP through supportive leadership, time allocation, and access to evidence resources.

**Abstract:**

**Background/Objectives**: Evidence-based practice (EBP) is essential to high-quality nutrition care. Despite its critical importance, its use among clinical dietitians in Saudi Arabia remains poorly understood. This study aims to examine the behavioral factors of EBP engagement among Saudi clinical dietitians using the Capability–Opportunity–Motivation–Behavior (COM-B) model. **Methods:** This cross-sectional mixed-methods study was conducted in fall 2023 among 82 licensed clinical dietitians (LCDs). Participants completed a self-administered questionnaire including 19 items adapted from the Health Sciences Evidence-Based Practice (HS-EBP) instrument and mapped to the COM-B domains. Qualitative data were obtained from open-ended survey questions completed by a separate sample of LCDs (*n* = 12) and analyzed using inductive thematic analysis. **Results:** Descriptive analysis showed higher mean scores for capability (7.49 ± 2.76) and motivation (7.50 ± 2.57) than for opportunity (6.61 ± 2.74) and behavioral engagement (6.09 ± 1.25). All COM-B domains demonstrated excellent internal consistency (Cronbach’s α = 0.857–0.959) and were significantly intercorrelated (*p* < 0.001). Multiple regression analysis indicated that motivation (β = 0.563, *p* = 0.001) and opportunity (β = 0.290, *p* = 0.002) independently predicted behavioral engagement (adjusted R^2^ = 0.681), while capability was not independently associated. Qualitative findings identified professional motivation and research literacy as facilitators, and time pressure and administrative workload as key barriers. **Conclusions:** EBP engagement among Saudi clinical dietitians is strongly influenced by motivational and organizational factors rather than by knowledge or skills.

## 1. Introduction

Evidence-based practice (EBP) is recognized as a core professional competency that supports safe, effective, and patient-centered care across health professions [1]. Major professional associations, such as the Academy of Nutrition and Dietetics [2] and the World Health Organization [3], have made EBP a core component of safe, responsible, and client-driven nutrition care. The application of EBP is essential in dietetics to remain responsive to patient needs and clinical settings, as nutrition guidelines continue to evolve in response to new scientific evidence [4,5].

In recent years, there has been a significant push to adopt EBP in Saudi Arabia. National healthcare transformations increasingly emphasize evidence-based clinical decision-making and the standardization of nutrition services across public and private healthcare settings [6,7]. Despite the policy commitment, there is a significant gap in understanding how clinical dietitians perceive and use EBP in everyday practice in the region [8]. Most regional studies conducted to date have employed a theoretical approach to assess knowledge or attitudes, without using behavioral frameworks to explain the uneven adoption of EBP among practitioners [9]. However, awareness alone is insufficient to close the gap between knowledge and practice; behavioral change depends on understanding the barriers and facilitators to EBP adoption in routine clinical settings [10]. Moreover, evidence from multiple health professions suggests that the implementation of EBP depends on the interaction between clinicians’ psychological skills, the opportunities available within organizational and practice settings, and motivation shaped by professional beliefs and intentions [11,12].

Thus, translating evidence into sustained practice is a persistent challenge in clinical settings, which require theory-driven strategies to close the knowledge-to-practice gap [13]. The Capability, Opportunity, Motivation, Behavior (COM-B) theory provides a comprehensive framework for understanding behavior [10]. It proposes that behavior occurs only when individuals have sufficient capability and opportunity and when motivation outweighs competing influences [10,14]. Thus, using a COM-B-informed approach to increase the uptake of EBP in nutritional care would necessitate that dietitians possess the knowledge and skills to interpret research evidence (capability), sufficient time, access to resources, and collegial support (opportunity), together with professional commitment to evidence-informed decision-making (motivation) [10,15].

While the COM-B model has been applied previously to explore behavioral influences on dietitians’ professional practice, such as weight management and counseling behaviors, and to inform behavior change training interventions [16,17], its application to examine routine EBP engagement within the field of dietetics remains limited. The Health Sciences Evidence-Based Practice (HS-EBP) questionnaire provides a validated measure of EBP beliefs and behaviors across health professions [18,19]. To our knowledge, it has not previously been systematically mapped onto the COM-B theory framework or culturally adapted for Middle Eastern contexts. Integrating the COM-B framework with the HS-EBP instrument, therefore, offers a theory-driven approach to explain how psychological, contextual, and motivational factors collectively shape EBP engagement among clinical dietitians [20].

Behavioral models have increasingly been applied to understand professional practice in healthcare, including dietetics. The COM-B framework has been used to examine behavioral determinants of dietitians’ professional practice, including counseling behavior and weight management practice, highlighting the importance of motivation and environmental opportunity in shaping clinical behavior [16,17]. Similarly, in broader healthcare contexts, behavioral and implementation science frameworks have been applied to explain variation in evidence-based practice and to inform strategies that support the integration of evidence into routine clinical care [10,13]. These applications demonstrate the relevance of behavioral theory-based approaches for understanding and improving EBP engagement across healthcare professions.

To summarize, three critical gaps in the existing literature can be identified. First, quantitative theory-informed studies examining the behavioral determinants of EBP engagement among clinical dietitians remain scarce. Second, existing EBP measurement tools have rarely been aligned with behavioral theory to explain variation in EBP behavior across clinical settings. Third, the relative roles of capability, motivation, and opportunity in predicting routine EBP engagement remain underexplored in this context, thereby limiting the evidence base for designing targeted, sustainable implementation interventions. Addressing these gaps through a theoretically informed mixed-methods approach will provide actionable evidence to support dietetic education, institutional capacity-building initiatives, and health policy development. Accordingly, this study aimed to investigate the behavioral determinants of EBP among clinical dietitians in Saudi Arabia using the COM-B model.

## 2. Materials and Methods

### 2.1. Study Design and Theoretical Framework

This study adopted a cross-sectional mixed-methods design guided by the COM-B model [10]. EBP among clinical dietitians was defined as the target behavior and examined in relation to the COM-B domains of capability, opportunity, and motivation.

### 2.2. Participants and Setting

Clinical dietitians licensed to practice and currently working in Saudi Arabian healthcare institutions were recruited using institutional mailing lists and professional WhatsApp groups. Inclusion criteria included (i) a bachelor’s degree or higher in Clinical Nutrition, (ii) current involvement in patient care, and (iii) a valid Saudi Commission for Health Specialties licensure. Participation was entirely voluntary and anonymous, and no incentives were provided. Data was collected from September to December 2023.

### 2.3. Study Instrument

The quantitative component used the HS-EBP questionnaire, which assesses trans-professional EBPs across health fields [18]. While the original HS-EBP questionnaire contains 60 items, we selected and culturally adapted 19 items relevant to our study.

The selection of items was guided by theoretical alignment with the COM-B framework and relevance to clinical dietetic practice, rather than to replicate the full multidimensional structure of the original HS-EBP instrument. This approach enabled focused examination of behavioral determinants of EBP engagement while ensuring representation of each COM-B domain. Because the study objective was to examine behavioral associations rather than to develop or validate a new measurement instrument, the selected items were used to capture theoretically relevant constructs within the COM-B framework.

The selection and adaptation of questionnaire items were conducted in three stages: First, HS-EBP items were reviewed by two authors separately on their applicability to dietetic practice and their correspondence to the domains of the COM-B. Second, two senior faculty were consulted to confirm that items were clear, relevant, and culturally appropriate. All selected questionnaire items were then mapped to the COM-B domains. Two reviewers independently classified items by content. Three experts achieved consensus through discussion after reaching 89% initial inter-rater agreement. Mapping was verified against original HS-EBP domains. The final distribution of items was as follows: Psychological Capability (4 items), Reflective Motivation (5 items), Physical and Social Opportunity (7 items), and Behavioral Engagement (3 items). Psychological capability represented perceived EBP-related knowledge and cognitive skills; reflective motivation reflected intention and professional commitment to apply EBP; physical and social opportunity captured organizational and contextual enablers; and behavioral engagement indicated routine use of research evidence in practice. All items were measured on 10-point Likert scales (1 = strongly disagree, 10 = strongly agree). To maintain uniformity in the directionality, reverse-coded items were recorded. Prior to data collection, the adapted questionnaire was pilot tested with five LCDs to assess clarity, comprehensibility, and contextual relevance. Participants confirmed that the items were clear and understandable and no modifications were required.

Correspondence at the detailed item level is provided in Supplementary Appendix S1.

### 2.4. Quantitative Data Analysis

All quantitative data analyses were performed using IBM SPSS Statistics version 29.0 (IBM Corp., Armonk, NY, USA) Mean scores were calculated for each COM-B domain. Internal consistency reliability was assessed using Cronbach’s α (≥0.70 acceptable). Since Shapiro–Wilk tests indicated significant deviations from normality across all domains (*p* < 0.05), non-parametric procedures were employed for bivariate analysis. Spearman’s rank correlation was used to assess associations between domains.

Despite the non-normality of the observed variables, multiple linear regression was conducted to examine independent predictors of EBP engagement, as the method is generally robust to violations of normality when appropriate diagnostic procedures are applied [21]. Thus, residual normality, homoscedasticity, variance inflation factors, and heteroscedasticity-consistent standard errors (HC3) were examined as part of the diagnostic checks. Regression analysis was retained as the analytic approach because the primary aim was to examine theoretical associations between COM-B domains, and HC3 standard errors were applied to provide robust estimates despite deviations from normality.

### 2.5. Qualitative Data Collection and Analysis

A separate survey with five open-ended questions and demographic items was administered to a subset of enrolled participants, yielding qualitative data on barriers and facilitators to the routine use of EBP in clinical settings. Responses were analyzed using Braun and Clarke’s six-phase thematic analysis framework [22]. Two researchers independently coded the text, and disagreements were resolved through discussion. Themes were aligned with the COM-B domains.

### 2.6. Integration of Quantitative and Qualitative Strands

A convergent parallel mixed-methods design was used, with quantitative and qualitative data analyzed concurrently and integrated at the interpretation stage using triangulation across COM-B domains.

## 3. Results

### 3.1. Participant Characteristics

Eighty-two clinical dietitians completed the quantitative survey, and 12 dietitians participated in the qualitative survey. Demographic and professional characteristics for both samples are summarized in Table 1. Quantitative samples were predominantly female (86.6%; *n* = 71), aged 22–31 years (69.5%; *n* = 57), held a bachelor’s degree (65.9% (*n* = 54) versus 29.3% (*n* = 24) with a master’s degree), and worked in government hospitals (79.3%; *n* = 65). Participants’ clinical experience varied, with 43.9% (*n* = 36) reporting having less than three years of experience and 22.0% (*n* = 18) having three to five years, while 34.1% (*n* = 28) had more than five years.

The qualitative subsample was all female (100%; *n* = 12) and represented a more experienced cohort, with 66.7% (*n* = 8) having more than 5 years of clinical practice. Ages ranged from 22 to 51 years, with 50% (*n* = 6) aged 22–31 years and 33.3% (*n* = 4) aged 32–41 years. Most of them held a bachelor’s degree (83.3%, *n* = 10) and worked in governmental settings (83.3%, *n* = 10), which reflects the distribution in the quantitative sample.

### 3.2. Quantitative Findings

#### 3.2.1. Levels of COM-B Domains

Descriptive statistics for the four COM-B domains are presented in Table 2. Psychological Capability (median = 8.50, IQR = 6.31–9.50) and Reflective Motivation (median = 8.35, IQR = 7.05–9.20) showed high test scores. Physical and Social Opportunity showed a slightly lower median score with wider dispersion (median = 7.57, IQR = 4.75–8.86), and Behavioral Engagement (median = 6.33, IQR = 5.67–8.92) showed relatively lower scores. Internal consistency reliability was excellent across all four domains, with Cronbach’s α coefficients ranging from 0.857 (Behavioral Engagement) to 0.959 (Psychological Capability), all exceeding the generally accepted 0.70.

#### 3.2.2. Inter-Relationships Between COM-B Domains

Spearman’s rank correlation was used to examine relationships among the four COM-B domains (Table 3). Psychological Capability and Reflective Motivation showed a robust correlation (ρ = 0.863, 95% CI [0.80, 0.91]). Physical and Social Opportunity also demonstrated a strong positive correlation with both Behavioral Engagement (ρ = 0.661, 95% CI [0.52, 0.77]) and Reflective Motivation (ρ = 0.669, 95% CI [0.53, 0.78]). Opportunity and capability were strongly and positively correlated (ρ = 0.602, 95% CI [0.45, 0.72]).

#### 3.2.3. Predictors of Behavioral Engagement

Multiple linear regression analysis was used to examine Capability, Motivation, and Opportunity as simultaneous predictors of Behavioral Engagement (Table 4). The overall model was statistically significant, F (3, 78) = 58.1, *p* < 0.001, and accounted for 68.1% of the variance in Behavioral Engagement (adjusted R^2^ = 0.681). Reflective Motivation (β = 0.563, 95% CI [0.233, 0.893], *p* = 0.001) and Physical and Social Opportunity (β = 0.290, 95% CI [0.112, 0.468], *p* = 0.002) were independent predictors of Behavioral Engagement. However, Psychological Capability did not significantly predict Behavioral Engagement (β = 0.000, 95% CI [−0.288, 0.288], *p* = 0.999). Variance inflation factor (VIF) values (range = 6.7–8.2) indicated multicollinearity among the COM-B predictors (Table 4). This reflects shared variance between domains included in the regression model.

### 3.3. Qualitative Findings

A 90% intercoder agreement was achieved for the five open-ended questions that were analyzed using Braun and Clarke’s six-phase thematic analysis framework. Qualitative findings from open-ended responses identified themes related to motivation, capability, and opportunity regarding engagement with EBP. Representative quotations are presented in Table 5.

#### 3.3.1. Motivation Domain

Participants highlighted several motivational factors for engaging in EBP, including the desire to improve patient outcomes, support clinical decision-making, fulfill professional responsibilities, address ethical considerations, and provide evidence-based advice to maintain trust and credibility with patients and colleagues. Additionally, some participants mentioned personal curiosity and a commitment to lifelong learning as reasons for engaging with EBP.

#### 3.3.2. Capability Domain

Participants reported confidence in their ability to read, interpret research, and locate relevant evidence when needed. They frequently cited the use of clinical protocols and guidelines to apply evidence in routine practice. Database searching and continuing education were also reported to support EBP engagement.

#### 3.3.3. Opportunity Domain

Participants identified several opportunity-related factors that facilitated engagement with EBP, such as access to evidence resources and databases, supportive leadership, and collaborative team environments that enabled discussion and the sharing of new evidence. Conversely, barriers included limited time and administrative support.

#### 3.3.4. Suggested Institutional and Policy Support

Participants reported a need for institutional and system-level support for EBP. Reported strategies included regular training workshops, improved access to scientific journals through institutional subscriptions, organizational policies that prioritize the use of evidence in clinical roles, and collaboration between academic institutions and clinical practice settings for knowledge exchange and shared learning.

## 4. Discussion

In the current study, clinical dietitians reported adequate EBP knowledge and skills. However, these alone were insufficient to support routine behavioral practice. This observation was consistent with findings from other healthcare professions in Saudi Arabia, such as nursing, that demonstrate that EBP knowledge and positive attitudes do not typically translate into sustained EBP use, particularly in settings with limited organizational support [23,24]. For example, a recent scoping review of nursing studies found that the routine implementation of EBP among clinicians was constrained by limited time, insufficient resources, and weak organizational support, despite positive attitudes toward EBP [12]. Similarly, recent reviews in the nutrition literature have also identified a persistent gap between knowledge, attitudes, and practices in dietetics. Many practitioners showed positive attitudes and confidence towards EBP, yet their application of evidence in routine practice remains inconsistent [25,26]. This persistent gap between knowledge, attitude, and practice is explained by behavioral theories such as the COM-B model, which asserts that capability must be supported by sufficient motivation and opportunity for evidence to be translated into action in real-world clinical settings [9,14,20].

Furthermore, our findings indicate that EBP was more strongly associated with motivational and contextual factors than with psychological capability among clinical dietitians. In contrast to previous dietetics research, which shows that higher educational attainment and more recent training are associated with greater objective and perceived knowledge of EBP [27], the current study has focused mainly on EBP preparedness and self-assessed competence. Nevertheless, our findings extend the literature by demonstrating that reflective motivation and physical and social opportunity are independently associated with EBP engagement among clinical dietitians. These findings support calls to move beyond defining professional competence solely in terms of knowledge and technical skills, and to recognize the role of motivational and organizational contexts in shaping routine clinical practice [28,29].

Compared with commonly used approaches that focus primarily on knowledge, attitudes, and perceived barriers, the COM-B model explicitly links capability, opportunity, and motivation to behavior, providing a behavioral explanation for why positive attitudes and adequate knowledge may not translate into routine EBP engagement [9,10,13]. Traditional knowledge–attitude–practice and barrier-focused approaches typically identify determinants of evidence use but do not explicitly model how these determinants interact to generate behavior. In contrast, the COM-B framework conceptualizes behavior as the result of interacting psychological and contextual conditions, enabling a more integrated understanding of implementation challenges and providing a clearer basis for designing targeted interventions [10,11,13]. Recent workforce research supports our findings, indicating that clinical nutrition practices differ significantly across various institutional settings and professional roles [29,30]. These practices are primarily shaped by hospital infrastructure, employment conditions, and role responsibilities rather than by education alone [29].

Together, the quantitative and qualitative findings from the present study help explain how these patterns operate in practice. Motivation was commonly associated with professional responsibility and commitment to patient care, while opportunity was restricted by time pressure, administrative workload, and organizational rigidity. These findings indicate that EBP engagement is conditional rather than consistently part of professional practice, becoming more likely when enabling conditions such as access to resources, opportunities for professional dialog, and organizational flexibility are present. In this context, the absence of a significant link between psychological capability and EBP engagement may reflect a functional threshold rather than a lack of competence. Once foundational EBP knowledge is established, engagement appears to be primarily influenced by motivational and organizational factors that facilitate the application of existing capabilities in practice [10,13,31].

When interpreted through the COM-B framework, patterns in the findings suggest that challenges in sustaining EBP may arise not from deficits in knowledge or attitudes, but at points where research evidence conflicts with established routines. Although EBP engagement was assessed as a composite construct, the observed pattern of associations, together with qualitative insights, indicates that habitual practice may override reflective, evidence-informed intentions in settings with limited organizational support. This interpretation highlights habit disruption as a plausible mechanism influencing sustained EBP engagement and reinforces the view that EBP represents cognitively and ethically engaged professional behavior rather than procedural compliance [10].

Accordingly, the lack of an independent association between psychological capability and EBP engagement should be interpreted cautiously and may reflect shared variance with motivation, given the moderate multicollinearity observed among the COM-B domains, rather than the absence of relevance of capability to EBP engagement. The findings have important implications for practice, workforce development, and education. Traditional approaches to improving EBP have emphasized individual-level training to enhance research knowledge and appraisal skills. However, the findings of the current study underscore the need to go beyond training to foster reflective motivation by strengthening professional identity, reinforcing ethical responsibility, and emphasizing the clinical relevance of evidence use. Consistent with previous research, leadership support and organizational feedback mechanisms play a central role in shaping clinicians’ engagement with EBP [11,12]. At the organizational level, the influence of physical and social opportunity highlights the need for system-level structures that actively support evidence use. Organizational conditions such as access to scientific resources and leadership expectations shape the extent to which EBP can be implemented in routine care [32]. Evidence from implementation science further indicates that integrating evidence use into routine workflows and fostering supportive practice environments are associated with higher EBP engagement, even among motivated clinicians [12,13]. From a policy and workforce perspective, the current study’s findings indicate that responsibility for EBP implementation should not rest solely with individual clinicians [32]. Instead, health systems and governing bodies must address modifiable system-level levers such as workload allocation, protected time for evidence use, access to scientific resources, and leadership expectations to enable sustained EBP engagement [33]. Policies that embed EBP within routine role expectations, performance frameworks, and organizational quality structures may be more effective than education-only strategies [33].

To our knowledge, few studies have examined the behavioral determinants of EBP engagement among clinical dietitians using the COM-B framework, particularly within the Saudi healthcare context. From a practical perspective, the COM-B model provides a structured framework for identifying modifiable determinants of EBP engagement among clinical dietitians. In the present study, reflective motivation and physical and social opportunity were significant predictors of EBP engagement, indicating that implementation efforts should focus on reinforcing clinicians’ motivation and ensuring that workplace environments support evidence use. This approach enables healthcare organizations to move beyond education-only strategies and to address behavioral and contextual factors that influence whether evidence is applied in routine clinical practice.

Despite these significant implications, several limitations of the current study should be acknowledged. First, the sample consisted primarily of clinical dietitians working in governmental hospitals in Saudi Arabia, which may limit generalizability to other healthcare settings. However, given the leading role of governmental hospitals within the national healthcare system, the sample provides a relevant perspective on current practice. Second, the use of self-administered questionnaires may have introduced reporting or social desirability bias, although anonymity was ensured to reduce these effects. Third, the cross-sectional design limits causal interpretation of the findings. In addition, the relatively small sample size may limit the generalizability of the findings and reduce the stability of regression estimates. Finally, the qualitative subsample was small and included only female participants, which may limit perspective diversity and theoretical saturation. Nevertheless, the use of a validated instrument, a mixed-methods design, and strong internal consistency support the reliability of the findings.

Full psychometric validation of the shortened HS-EBP instrument, including factor analysis and construct validity testing, was not conducted. Although item selection was guided by expert review and theoretical alignment with the COM-B framework, some degree of construct overlap between domains may be present. Furthermore, multicollinearity among COM-B domains may have influenced the stability and interpretability of individual regression coefficients, and the non-significant association observed for psychological capability should be interpreted cautiously, as it may reflect shared variance with motivational factors rather than the absence of a meaningful relationship.

## 5. Conclusions

In conclusion, engagement with EBP among clinical dietitians in Saudi Arabia was more strongly associated with reflective motivation and physical/social opportunity than with psychological capability. These findings suggest that EBP engagement is shaped by organizational conditions and professional motivation, and may be difficult to sustain when time, resources, and institutional expectations are limited. The COM-B model offers a useful framework to guide theory-informed workforce and organizational strategies that support routine evidence use. However, the findings should be interpreted in light of the cross-sectional design, self-reported measures, and the relatively small sample. Future research should evaluate theory-informed interventions targeting modifiable motivational and opportunity factors and assess their impact on sustained EBP engagement across diverse clinical settings. Larger studies with more diverse and representative samples are needed to confirm and extend these findings.

## Figures and Tables

**Table 1:** Participant characteristics for quantitative (*n* = 82) and qualitative (*n* = 12) samples.

Characteristic	Category	Quantitative (*n* = 82)	Qualitative (*n* = 12)
Gender	Female	71 (86.6%)	12 (100%)
	Male	11 (13.4%)	-
Age (years)	22–31	57 (69.5%)	6 (50%)
	32–41	16 (19.5%)	4 (33.3%)
	42–51	9 (11.0%)	2 (16.7%)
Education level	Bachelor	54 (65.9%)	10 (83.3%)
	Master	24 (29.3%)	1 (8.3%)
	PhD	4 (4.9%)	1 (8.3%)
Years of experience	<3 years	36 (43.9%)	1 (8.3%)
	3–5 years	18 (22.0%)	3 (25.0%)
	>5 years	28 (34.1%)	8 (66.7%)
Work sector	Governmental	65 (79.3%)	10 (83.3%)
	Private	17 (20.7%)	2 (16.7%)

**Table 2:** Descriptive statistics of COM-B constructs, internal consistency, and interpretation among participating dietitians (*n* = 82).

COM-B Domain	Mean ± SD	Median	IQR (Q1–Q3)	Cronbach’s α	Interpretation
Capability (Psychological)	7.49 ± 2.76	8.50	6.31–9.50	0.959	Excellent
Motivation (Reflective)	7.50 ± 2.57	8.35	7.05–9.20	0.936	Excellent
Opportunity (Physical/Social)	6.61 ± 2.74	7.57	4.75–8.86	0.944	Excellent
Behavioral Engagement	6.09 ± 1.25	6.33	5.67–8.92	0.857	Good

Note: All items were rated on 10-point Likert scales (1 = strongly disagree, 10 = strongly agree). All domains deviated significantly from normality (Shapiro–Wilk, *p* < 0.05). Reliability interpretation follows conventional benchmarks: α ≥ 0.90 = excellent; α ≥ 0.80 = good; α ≥ 0.70 = acceptable.

**Table 3:** Correlations between COM-B domains (Spearman’s ρ, *n* = 82).

Variable	1. Capability	2. Motivation	3. Opportunity	4. Behavior
1. Capability	-	-	-	-
2. Motivation	0.863 ***	-	-	-
3. Opportunity	0.602 ***	0.669 ***	-	-
4. Behavior	0.480 ***	0.554 ***	0.661 ***	-

Note: *** *p* < 0.001 for all correlations. Interpretation: weak < 0.30, moderate 0.30–0.49, strong 0.50–0.69, very strong ≥ 0.70.

**Table 4:** Multiple linear regression model predicting Behavioral Engagement with EBP among participating dietitians (*n* = 82).

Predictor	β (HC3)	95% CI	*p* (HC3)
Intercept	0.806	0.103–1.508	0.025
Capability (Psychological)	0.000	−0.288–0.288	0.999
Motivation (Reflective)	0.563	0.233–0.893	0.001
Opportunity (Physical/Social)	0.290	0.112–0.468	0.002

Note: Model summary: F(3, 78) = 58.1, *p* < 0.001; Adjusted R^2^ = 0.681; VIF range = 6.7–8.2. HC3 = heteroscedasticity-consistent standard errors (type 3).

**Table 5:** Frequency of key qualitative themes mapped to COM-B domains (*n* = 12).

COM-B Domains	Sub-Themes	Representative Quotation	*n* (%)
Motivation	Improving patient outcomes	“To improve patient outcomes and make well-informed clinical decisions.”	9 (75%)
	Professional responsibility/ethics	“Providing evidence-based advice builds confidence and trust with patients and colleagues.”	8 (67%)
	Personal curiosity and lifelong learning	“I read more studies not only for work but to develop myself and my knowledge.”	7 (58%)
Capability	Reading and interpreting research	“Reading research and knowing where to find the evidence.”	10 (83%)
	Following clinical protocols and guidelines	“I rely on hospital protocols that are already based on scientific research.”	6 (50%)
	Database search and continuous education	“Searching databases and attending scientific conferences help me apply EBP.”	5 (42%)
Opportunity	Access to evidence resources	“Access to valid resources and databases makes it easier to apply EBP.”	8 (67%)
	Supportive leadership and collaborative culture	“Team cooperation and leadership support help share and discuss new evidence.”	7 (58%)
	Time and workload barriers	“Limited time and administrative tasks make it harder to focus on research.”	9 (75%)
Suggested Supports	Training workshops and short courses	“Provide regular workshops and short courses to exchange experience and update knowledge.”	10 (83%)
	Policy support and access to journals	“Memberships for scientific journals and supportive institutional policies are needed.”	8 (67%)
	National collaboration between academic and clinical sectors	“Create collaboration between academic and clinical dietitians to share evidence.”	6 (50%)

Note: Percentages indicate frequency of mentions in all twelve interviews. Each participant could contribute to multiple themes within and across domains.

## Data Availability

The data are available on reasonable request from the corresponding author.

## Supplementary Materials

The following supporting information can be downloaded at: https://www.mdpi.com/article/10.3390/healthcare14070893/s1, Supplementary Appendix S1: Evidence-Based Practice Survey Items (Adapted from HS-EBP and Mapped to COM-B).

## Abbreviations

The following abbreviations are used in this manuscript:

EBP	Evidence-Based Practice
COM-B	Capability, Opportunity, Motivation–Behavior
HS-EBP	Health Sciences Evidence-Based Practice
LCD	Licensed Clinical Dietitian
