# Supplementary material for: Factors Influencing Evidence-Based Practice Engagement Among Clinical Dietitians: A Mixed-Methods Study Using the COM-B Model
Source: Healthcare (Basel). 2026 Mar 31;14(7):893. doi: 10.3390/healthcare14070893 (PMC13073441; doi:10.3390/healthcare14070893)
Supplement: Supplementary file 1 [file healthcare-14-00893-s001.zip › healthcare-4114740-supplementary.pdf]

Supplementary Appendix S1. Evidence-Based Practice Survey Items (Adapted from HS-EBP and Mapped to COM-B)

Instructions to participants

This section aims to assess your opinion concerning aspects related to Evidence-Based Practice (EBP). Please rate your level of agreement with each statement on a scale from 1 to 10, where 1 corresponds to the lowest agreement and 10 corresponds to the highest agreement.

Response options (all items):

1 2 3 4 5 6 7 8 9 10

Section A. Capability (Psychological)

(COM-B domain: Capability)

C1. I am able to carry out an effective search of scientific literature in electronic databases.

C2. I know the different designs of scientific studies that will enable me to answer my doubts or my questions.

C3. I am capable of interpreting the practical implications of statistical results.

C4. I usually assess the quality of the methodology used in the research studies I find.

Section B. Motivation (Reflective)

(COM-B domain: Motivation)

M1. I consider it motivating to apply EBP.

M2. I am willing to change my professional practice when scientific evidence supports it.

M3. I intend to increase the use of scientific evidence in my professional practice.

M4. EBP must play a positive role in my professional practice.

M5. Applying EBP is among my professional priorities.

Section C. Behavioral Engagement

(COM-B domain: Behavior)

B1. I incorporate up-to-date research results to solve problems in my practice.

B2. When research results do not agree with my usual practice, I change it to incorporate them.

B3. I repeat interventions that have given me good results even if not supported by research. (reverse-coded)

Section D. Opportunity (Physical andSocial)

(COM-B domain: Opportunity)

O1. I use exchanges of opinions with other professionals in my daily practice.

O2. When facing situations not resolved by research, I ask for expert opinions.

O3. I can access resources related to scientific evidence in my workplace.

O4. In my workplace, there are documents that guide interventions toward EBP.

O5. At work, there are spaces to share and discuss scientific research results.

O6. EBP application is encouraged or rewarded in my workplace.

O7. Changing established patterns of practice in my workplace is easy.

Scoring note

All items were rated on a 10-point Likert scale (1 = lowest agreement; 10 = highest agreement). Higher scores indicate stronger endorsement of EBP beliefs/conditions or greater engagement with EBP behaviors. Item B3 was reverse-coded prior to computing domain scores so that higher scores consistently reflect greater EBP engagement.
